# A high-throughput protocol for mutation scanning of the *BRCA1 *and *BRCA2 *genes

**DOI:** 10.1186/1471-2407-11-265

**Published:** 2011-06-24

**Authors:** Heather L Hondow, Stephen B Fox, Gillian Mitchell, Rodney J Scott, Victoria Beshay, Stephen Q Wong, kConFab Investigators, Alexander Dobrovic

**Affiliations:** 1 Molecular Pathology Research and Development Laboratory, Department of Pathology, Peter MacCallum Cancer Centre, Locked Bag 1, A'Beckett St, Melbourne, Victoria, 8006, Australia; 2 Department of Pathology, The University of Melbourne, Parkville, Victoria, 3010, Australia; 3 Familial Cancer Centre, Peter MacCallum Cancer Centre, Locked Bag 1, A'Beckett St, Melbourne, Victoria, 8006, Australia; 4 School of Biomedical Sciences, University of Newcastle, New South Wales, 2308, Australia

## Abstract

**Background:**

Detection of mutations by DNA sequencing can be facilitated by scanning methods to identify amplicons which may have mutations. Current scanning methods used for the detection of germline sequence variants are laborious as they require post-PCR manipulation. High resolution melting (HRM) is a cost-effective rapid screening strategy, which readily detects heterozygous variants by melting curve analysis of PCR products. It is well suited to screening genes such as *BRCA1 *and *BRCA2 *as germline pathogenic mutations in these genes are always heterozygous.

**Methods:**

Assays for the analysis of all coding regions and intron-exon boundaries of *BRCA1 *and *BRCA2 *were designed, and optimised. A final set of 94 assays which ran under identical amplification conditions were chosen for *BRCA1 *(36) and *BRCA2 *(58). Significant attention was placed on primer design to enable reproducible detection of mutations within the amplicon while minimising unnecessary detection of polymorphisms. Deoxyinosine residues were incorporated into primers that overlay intronic polymorphisms. Multiple 384 well plates were used to facilitate high throughput.

**Results:**

169 *BRCA1 *and 239 *BRCA2 *known sequence variants were used to test the amplicons. We also performed an extensive blinded validation of the protocol with 384 separate patient DNAs. All heterozygous variants were detected with the optimised assays.

**Conclusions:**

This is the first HRM approach to screen the entire coding region of the *BRCA1 *and *BRCA2 *genes using one set of reaction conditions in a multi plate 384 well format using specifically designed primers. The parallel screening of a relatively large number of samples enables better detection of sequence variants. HRM has the advantages of decreasing the necessary sequencing by more than 90%. This markedly reduced cost of sequencing will result in *BRCA1 *and *BRCA2 *mutation testing becoming accessible to individuals who currently do not undergo mutation testing because of the significant costs involved.

## Background

Inactivating germline mutations in the *BRCA1 *and *BRCA2 *tumour suppressor genes dramatically escalates the risk of developing breast and/or ovarian cancer by up to 20 fold [1-4]. Due to the highly penetrant nature of germline mutations within *BRCA1 *and *BRCA2*, it is of importance to identify a woman as being a carrier of a mutation as early intervention measures including breast screening and prophylactic bilateral salphingo-oophorectomy or mastectomy can be offered [5]. More recently, it has been recognised that *BRCA1 *or *BRCA2 *mutant tumours are sensitive to PARP inhibitors and thus rapid and inexpensive *BRCA1 *and *BRCA2 *testing may be of direct clinical utility [6].

*BRCA1 *and *BRCA2 *are very large genes. *BRCA1 *has 24 exons (22 of which are protein coding) that code for a 1863 amino acid protein while *BRCA2 *has 27 exons (26 coding) that code for a 3418 amino acid protein. Currently, Sanger sequencing is considered as the gold standard for identification of sequence variants within *BRCA1 *and *BRCA2*. However, scanning methods have been often employed in order to reduce costs and improve turn around time. The major disadvantage of most scanning methods is that they require post-PCR product manipulation which, in addition to the increased workload, also carries the potential risks of sample misidentification and contamination [7,8].

High Resolution Melting (HRM) is a post-PCR method which enables the detection of sequence variations within an amplified region of DNA. Using saturating concentrations of a fluorescent dye which specifically intercalates with double stranded DNA, the denaturation behavior of an amplified region of DNA can be analysed. The dye dissociates from the double stranded DNA as it denatures into single stranded DNA and thus the melting can be monitored using the decrease in fluorescence. Heterozygous sequence variations are readily detected due to the formation of heteroduplexes between variant and wildtype strands that then have a characteristic early melting profile [9,10].

HRM has the major advantage over other pre-sequencing scanning methods in that it is performed in a "closed tube" system. This eliminates the risk of post-PCR product contamination during scanning while also reducing processing time (especially when the PCR and HRM are performed within the one instrument as in this study), resulting in improved turn around times. HRM has effectively replaced the previously most commonly used scanning method, denaturing high pressure liquid chromatography (DHPLC). It has better sensitivity and specificity for the detection of variants than DHPLC [7,8,11].

In this communication, we report the development of an HRM-based assay system for mutation detection within *BRCA1 *and *BRCA2 *using a 384 well plate format both to facilitate the detection of mutations and to enable high throughput scanning. As it is difficult to distinguish heterozygosity for SNPs from heterozygosity of other sequence variants, we have employed strategies for SNP minimisation within amplicons. We also introduced the use of deoxyinosine residues in HRM primers to enable the siting of primers over clinically insignificant intronic SNPs. The assay system is a robust *BRCA1 *and *BRCA2 *mutation scanning protocol that has had the most extensive validation so far reported.

## Results

### Amplicon design principles

Amplicons were selected in order to analyse the entire coding sequence and the intron-exon boundaries of *BRCA1 *and *BRCA2*. Typically, one amplicon was designed per exon. However, for longer exons or exons with more complex melting domains, two or more overlapping amplicons were chosen.

Extensive *in silico *analysis was performed in order to identify amplicons that would be suitable for both PCR and HRM. The DNA melting prediction software 'Poland' [12] was used to choose amplicons which preferably had a single melting domain. In some cases, amplicons with multiple domains were selected to keep the overall number of amplicons low. This was especially the case where the amplicon was short. Where double melting domains were unavoidable e.g. *BRCA2 *exon 11Q, the tested mutations were readily detectable (Additional file 1 Figure S1).

In germline DNA, all pathogenic *BRCA1 *and *BRCA2 *mutations will exist in heterozygous form giving rise to heteroduplexes that enhance variant detection by HRM. Previous HRM studies have reported 100% sensitivity for detection of heterozygous sequence variations in PCR products up to 435 bp [13-15]. All of the amplicons in this study were less than 405 bp. Although the homozygous genotypes for some of the polymorphisms were readily distinguishable e.g. the c.2612C>T in *BRCA1 *exon 11H (Additional file 2 Figure S2), others were not. This was not considered a problem as our aim was to identify heterozygous changes rather than to genotype existing high frequency polymorphisms.

### Primer design principles

In order for all of the assays to perform under the same PCR and HRM conditions, primers were designed to have an annealing temperature of 64-67°C using OligoCalc [16]. Primers were designed to minimise primer-dimer interference and were screened to ensure specificity to the target site using Amplify v3.1 [17]. Attention was paid to the 3' ends of the primers ensuring that they were neither too GC rich or AT rich.

Intronic polymorphisms within an amplicon decrease the specificity of HRM assays for mutation detection as they produce heterozygous melting profiles and thus require sequencing to distinguish them from true mutations. Primers were placed as close to exon boundaries as possible while still leaving at least 5 intronic bases to identify the most likely splicing mutations. This also assisted in keeping the amplicon size lower. However, where there were known or suspected pathogenic intronic variants close to the exons listed in the Breast Cancer Information Core Database (i.e. *BRCA1 *c.213-11T>G, *BRCA1 *c.5194-12G>A and *BRCA2 *c.426-12_8del5), the primers were moved further into the intron to enable the detection of these variants [18].

A critical consideration in primer design for mutation screening is that primers should not be placed over known polymorphisms, even if they are comparatively rare. This may lead to non-amplification of the polymorphic allele and coexisting pathogenic mutation if in a *cis *relationship with the polymorphism or false homozygosity of the inactivating mutation if it exists in a *trans *relationship with the polymorphic allele [19,20]. Care was thus taken to identify known single nucleotide polymorphisms (SNPs) using the Basic Local Alignment Search Tool (BLAST) [21]. Care was also taken to ensure that the primer sets did not non-specifically amplify the *BRCA1 *partial pseudogene where the homology extends into *BRCA1 *exon 2 [22].

**Figure 1:**
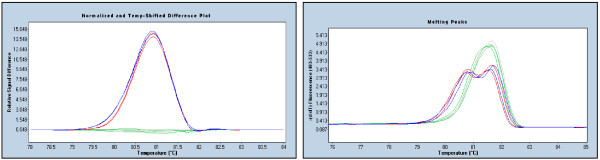
**Detection of both *BRCA2 *c.26delC mutant and wild type alleles where deoxyinosine is used at the c.-26G>A SNP**. The red and blue samples both contain the c.26delC deletion but differ according to their genotype for the *BRCA2 *c.-26G>A polymorphism. The red profile is homozygous wildtype for the polymorphism. The blue profile is heterozygous for the polymorphism. The left panel shows the difference curves where the baseline comprises both wildtype (grey) and heterozygous (green) genotypes. The right panel shows the corresponding melting peak curves. The difference curves are independent of the SNP meaning that both alleles are equally amplified using the deoxyinosine containing primers

When common or rare polymorphisms were present in an intronic region that was otherwise optimal for primer placement, we incorporated a deoxyinosine residue (dI) at the position of the polymorphism. Incorporating a dI into the primer at the site of the polymorphism allows equal amplification of both the wildtype and the variant allele [23]. During amplification, complementary bases will be inserted randomly producing a product that is indistinguishable from wildtype by HRM as dI will not form heteroduplexes regardless of which base it is paired with. Consistent with this, we found that incorporating a dI into the primer at the location of the polymorphism was a good strategy for eliminating the detection of the polymorphism while giving clean HRM profiles suitable for both mutation detection and downstream sequencing of mutations. Polymorphisms that were replaced by dI were, *BRCA2 *c.-26G>A (rs1799943) which flanks the coding region of exon 2, *BRCA2 *c.7806-14T>C (rs9534262) and *BRCA1 *c.80+13A>G. Amplification of both alleles of the polymorphism allows the detection of all coexisting mutations while masking the SNP. Figure 1 shows the example of detection of both the *BRCA2 *c.26delC mutant allele and the wild type allele where a dI in the primer has been used to mask the c.-26 G>A polymorphism.

### Protocol design

In HRM analysis, multiple samples for each amplicon should be screened at the same time in order to enable the ready detection of mutant sequences relative to multiple wild-type sequences as more samples act to dampen stochastic variation. As most samples are wildtype for any given amplicon, multiple samples will make it easy to distinguish a mutation from the wildtype variation. The use of 384 well plates facilitates the use of multiple samples. We chose a batch size of either 10 (6 plates) or 22 samples (12 plates). A known wild type control that was homozygous for all the common SNPs and a no-template control were also included. The amplicons were clustered into discrete areas on the plate to minimise the temperature variation for each amplicon (Additional file 3 Table S1 shows the layout).

Each primer set was initially tested using the standard mastermix and PCR and HRM program, after which primer sets requiring additional optimisation were tested using the simplified primer matrix. Only primer sets which produced specific products as assessed on an agarose gel and which both amplified efficiently and melted in an acceptable profile were used for mutation testing. Otherwise, new primer sets were designed for the region. In addition, primers needed to give good melt curves without any non-specific products.

The analysis was performed on a combined PCR and HRM instrument allowing us to analyze HRM data in the context of the PCR amplification information. During analysis of the amplification for each amplicon, any outlying late amplifying replicates that were often associated with false positive alterations in melting profile were removed from the analysis.

### Optimizing the set of HRM amplicons against a panel of known sequence variants

In the final protocol, 36 amplicons were used to analyze *BRCA1 *while 58 amplicons were used to analyze *BRCA2 *(Additional file 4 Table S2). Initially, a provisional set of assays were designed. For these, a large panel of mutation positive and other sequence variant controls were used to test the ability of the amplicons to identify sequence variants. The controls were analysed alongside multiple wild type controls derived from women that had previously tested negative for sequence variants.

Some amplicons required further optimisation of the primer concentration which was determined using a range of concentrations of the forward and reverse primers in a primer matrix [24]. Agarose gels were run for each primer set to ensure that the correct size product was produced and that there were no non-specific products or primer dimers formed.

Mutation detection was carried out using all the three visualisations of the raw data (normalised and temperature shifted melting curves, normalised and temperature shifted difference curves, and the negative first derivative curves which give a melting peak curve). Melting peak curves are independent of normalisation and temperature shifting. However in general, the difference curve view was the most useful. Normalising the data at the pre-and post-melt phases of HRM allows samples to be directly compared.

Surprisingly since all heterozygous mutations might be expected to have pronounced heteroduplexes, some mutations, especially single base insertions and deletions, gave rise to more subtle shifts. Previous communications have also noted the difficulty in detecting single base insertion or deletion mutations [25,26]. We have found this to be most marked when the single base insertion or deletion exists within extended runs of a single nucleotide. Such variations create little Tm difference and are consequently more subtle with HRM. While we did experiment with additives such as DMSO with some success for difficult amplicons, we finally focused on amplicon design to maintain consistency in mastermix set-up.

**Figure 2:**
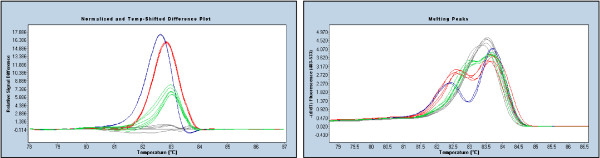
**Detection of different mutations within the same amplicon (*BRCA1 *11I region)**. The left panel shows the difference curves and the right panel shows the corresponding melting peak curves. All mutations produce obvious biphasic melting which is caused by the earlier melting of the heteroduplexes. The normalised and temperature-shifted difference plots in the left panel allow easy detection of the c.2863delTCATC (navy), c.2800C>T (red) and c.2885delA (green) relative to the wildtype HRM profile (grey). The melting peak curves in the right panel show that the c.2885delA is the most subtle mutation in that there is a minimal early melting heteroduplex component compared to the c.2863delTCATC and c.2800C>T mutations.

Figure 2 shows an example where the c.2885delA mutation in *BRCA1 *is less apparent than the other sequence variants, particularly when the melting peak curves are examined. Nevertheless the mutation is still obviously different from the wildtype. While single nucleotide changes and multiple base insertions and deletions are usually best detected using the normalised and temperature shifted melting curves, single base insertions and deletions are better detected on a difference curve relative to a wildtype control.

In some amplicons, certain mutations were initially not detected by the HRM assays. The exons that proved most problematic were *BRCA1 *exon 7 and *BRCA2 *exons 3, 11 and 15. For example, in *BRCA1 *exon 7, both the single base insertion c.329insA and single base deletion mutation c.302-2delA were not detected with the original amplicon. As a result, new amplicons were designed, optimised and validated in order to also detect the mutations and checked against the other mutation controls. For *BRCA2 *amplicon 11I, we were not initially able to detect the c.4512insT which inserted an extra T into a run of 6 Ts and thus included an extra amplicon (11Is) that covered a shorter region and was able to resolve this mutation (data not shown).

**Figure 3:**
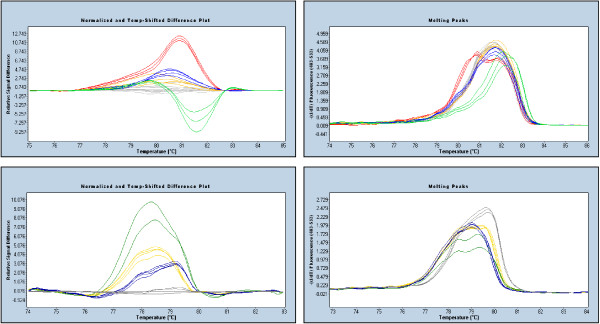
**The influence of amplicon choice on mutation detection**. The top panels show difference curves and melting peak curves for the original *BRCA1 *exon 7 amplicon which did not readily detect single base insertions or deletions. While the c.427G>T (red) and the c.314A>G (green) mutations are readily detectable in both visualisations, the pathogenic c.329insA (navy) and c.302-2delA (yellow) single base pair insertion and deletion mutations were difficult to detect as they melt like the wildtype controls (grey). The original amplicon was then divided into two overlapping amplicons. The bottom panels show difference curves and melting peak curves for *BRCA1 *amplicon exon 7A which detects the single base insertion and deletion. The c.329insA (navy) and c.302-2delA (yellow) clearly differ from the wildtype controls (grey) in the shorter amplicon. The *BRCA1 *c.314A>G (dark green) mutation is also readily detectable in both visualisations.

**Figure 4:**
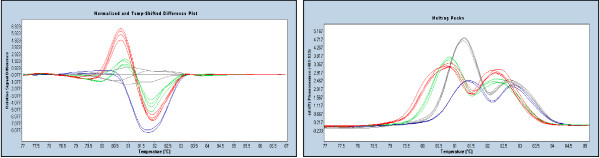
**Detection of an insertion within a long nucleotide repeat by HRM (*BRCA2 *exon 23)**. The mutation c.9097insA (green) is an insertion of an adenine nucleotide into an 8 adenine repeat. Like the other mutations here; c.9117G>A (red) and c.9117+1G>A (navy); it is readily distinguishable from the wildtype (grey) in this 264 bp amplicon.

Another solution was to divide problematic amplicons into 2 overlapping shorter amplicons making the melting profile within each amplicon less complex based on Poland analysis (Figure 3). By decreasing the size of the amplicon, we could detect all available mutations, including those that are located within repeat regions (Figure 4).

A large number of positive controls were tested for *BRCA1 *(184) and *BRCA2 *(256) (Additional file 5 Table S3). While most amplicons had multiple mutation controls (mean = 4.25), a few amplicons did not possess any mutation controls. These amplicons were *BRCA1 *exons 9 and 22, *BRCA2 *exons 11J2, exon 12, exon 26 and exon 27A. However, during subsequent blinded testing, mutations in all of these exons except 27A were detected. These and other new variants that were detected are detailed in Additional file 6 Table S4.

### Validation of the assays

In addition to the testing of the individual amplicons, two sets of validations using full HRM screens were performed. In these validations, we undertook a deliberate strategy of sequencing all reactions with minor deviations from wildtype i.e. those that were likely to be "false positives". As discussed, some pathogenic mutations can produce subtle changes to the melting behaviour. All reactions which were suspicious including those that had one aberrant replicate were sequenced. Thus, likely false positives were targeted for sequencing as the consequences of inadvertently missing a mutation are more serious than calling a false positive. We also sequenced all those variants that were likely to be SNPs.

In the first set, 266 samples which were sent for diagnostic testing by bidirectional Sanger sequencing were concurrently screened by the first version of our full HRM screen. The operator was blinded to the results of the mutation sequencing until the samples had been scored as to whether they contained variants by HRM. Subsequently the results of HRM and sequencing were compared. All variants including heterozygous SNPs were considered in the analysis.

22,797 amplicons were analysed and 7.5% were identified as requiring sequencing to identify the variant while 92.5% were considered to be wildtype. All 35 pathogenic mutations (13% of DNA samples) identified by sequencing were also detected by the HRM screening. The calculated sensitivity for the detection of heterozygous variants (including SNPs) was 99.8% (1595/1599) while the specificity was 99.4% (21,070/21,192). The positive predictive value was 92.9% while the negative predictive value was 99.98%. At this stage, the *BRCA1 *c.5074+3A>C, *BRCA2 *c.1834G>A, c.2971A>G, c.3807T>C and c.9458G>C variants were not detectable but subsequent amplicon redesign as used in the final protocol led to their detection and an effective sensitivity of 100%.

A second validation was performed by retrospectively re-screening 118 archival samples which were previously reported as negative for germline inactivating mutations within *BRCA1 *and *BRCA2 *with the final version of the protocol. These samples had previously undergone testing using a combination of testing procedures including protein truncation testing (PTT) and partial Sanger sequencing. Some of these samples had only been tested for one of the genes or had just undergone PTT testing for mutations in large exons. In the second validation, 11,092 fragments were analysed and 92.7% of amplicons were called wildtype. 7.3% of amplicons contained variants that required further investigation by sequencing. Sensitivity was 100% while specificity was 99.75%. The positive predictive value was 98.98% while the negative predictive value was 100%. The positive predictive value was decreased due to the deliberate strategy of also choosing reactions with minor deviations from wildtype that were likely to be "false positives" by HRM.

Once again, the positive predictive value was decreased due to the detection of "false positives" by HRM that were identified as wildtype by sequencing. Eighteen amplicons were "falsely positive" by HRM. Importantly, no amplicons were falsely negative by HRM.

### Mutations co-existing with polymorphisms

**Figure 5:**
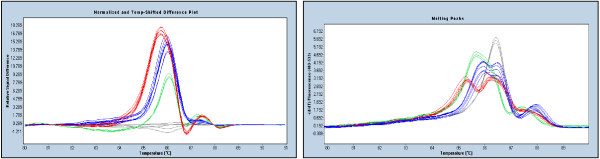
**Two variations within the same sample result in greater HRM differences. (*BRCA1 *exon 13)**. The left panel shows the difference plot where 3 samples have been compared to the wildtype controls (grey). The green profile represents the a heterozygote for common polymorphism c.4308T>C while the blue represents the mutation c.4327C>T. The presence of both sequence variants (red) results in greater instability than each individual sequence variation due to the double mismatch in heteroduplexes. The right panel with melting peak curves show the complex melting nature of two co-existing sequence variants (red).

In some cases, mutations may be present close to a common or rare polymorphism. De Juan *et al*. (2009) raised the theoretical concerns that pathogenic mutations which coexist with polymorphisms may distort the HRM curve and make it appear like a normal sequence [27]. We consider this extremely unlikely as greater instability (early melting) would be caused by multiple populations of heteroduplexes formed as a result of the two sequence variants. In support of this, we found several examples of a mutation coexisting with a common polymorphism and resulting in a greater difference between the melting curves of the doubly variant polymorphic sample and the wildtype (Figure 5).

### Detection of mutations adjacent to the 3' end of the primers

**Figure 6:**
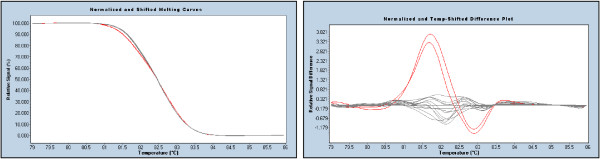
**Detection of a sequence variation immediately adjacent to the 3' end of a primer**. The class 1 SNP (*BRCA2 *c.1365A>G) (red) is readily detectable at the position immediately adjacent (1 base pair) to the 3' end of the reverse primer in this 232 bp amplicon (*BRCA2 *exon 10C).

It has been argued by some authors that mutations close to the primers are more difficult to detect than more centrally placed mutations. While it has been reported that the ability to detect mutations decreases as the distance between the primer and the mutation decreases [8,28], we were able to detect mutations immediately adjacent to the 3' end of the primer; an example being the *BRCA2 *c.1365A>G polymorphism in amplicon exon 10C (Figure 6). Other publications have confirmed that the location of the sequence variation within the amplicon does not affect the ability of HRM to detect the variation [9,29]. Van der Stoep *et al*. (2009) also reported detecting mutations up to 2 bp from the end of the primer [25].

## Discussion

Current dideoxy (Sanger) sequencing based methods for mutation identification are expensive and labor intensive even with a high degree of automation. Scanning methodologies can markedly reduce the amount of sequencing by identifying the limited number of PCR amplicons carrying variant sequences. High resolution melting has become the method of choice for scanning as it can be performed immediately after PCR amplification in a seamless closed tube protocol on a single instrument. It is also preferable to other scanning methods as it does not involve out-of-tube manipulation of the PCR product thereby reducing the potential for PCR contamination. Well designed and validated HRM assays detect practically every sequence variant [30].

Our aim was to develop a high throughput HRM protocol in order to enable *BRCA1 *and *BRCA2 *testing to be performed at considerably lower cost without compromising sensitivity or specificity. The cost reduction is significant as the number of amplicons to be sequenced is reduced by more than 90%. This involved sequencing of all variants. The majority of these turned out to be SNPs as expected. Within a research setting, costs could be further reduced by not sequencing those samples from amplicons spanning a common SNP where the melting curves were identical to those of the SNP.

An extensive validation process confirmed that HRM is a highly effective screen for the detection of heterozygous sequence variants within the *BRCA1 *and *BRCA2 *genes. Since pathogenic germline mutations always exist in a heterozygous state within germline DNA and homozygous germline variants only exist for non-clinically important variants such as polymorphisms, HRM is thus ideal for minimizing the burden of sequencing.

In the past, a combined pre-sequencing screening protocol of PTT and DHPLC followed by sequencing of amplicons found to possess sequence variants was often used. DHPLC was formerly the gold standard for variant scanning [31]. Comparative studies have shown that HRM has superior sensitivity and specificity than DHPLC [7,8]. Whereas HRM is able to detect all heterozygous mutations and some homozygous mutations within amplicons with complex melting domains, DHPLC sensitivity and specificity can be challenged within multiple melting domains. DHPLC also requires that the column temperature must also be empirically optimised for each amplicon whereas; we have developed HRM so that all amplicons undergo the same PCR and HRM reaction conditions. Thus, multiple HRM analyses can thus be carried out in parallel contrasting with DHPLC in which analyses are sequential.

HRM has been used as a rapid screen for *BRCA1 *and *BRCA2 *founder mutations. Dufresne et al (2006) first reported HRM for the detection of the 3 common Ashkenazi founder mutations using short amplicons [32]. We subsequently established that long amplicons could be successfully used for the Ashkenazi mutations [33]. This enabled the detection of other mutations, and this principle underlies the experimental design in this paper where it is often necessary to screen comparatively long stretches of DNA in order to efficiently scan the full coding sequence of the genes. Other populations studied for founder mutations by HRM include Spaniards, Greeks and Southern Chinese [27,34,35].

Full screening by HRM of exons including intron-exon boundaries has been reported for *BRCA1 *[25], and for *BRCA1 *and *BRCA2 *[26,36]. We have independently developed a comprehensive HRM protocol for full screening of exons and intron-exon boundaries for both genes. We aimed to design all the PCR assays so they could be amplified under identical conditions using a minimal amount of DNA in a high throughput (384 well) approach. Previous communications used 96 well plates and the layout and ergonomics were not described [26,36].

Each assay was tested using as many positive mutation controls as available to us. Where sequence variations were not immediately detectable as occurred in a few cases, new primers were optimised for the detection of that variation in addition to the other positive controls present within the amplicons. Extensive testing of the amplicons with known mutations and redesign where there were detection difficulties, resulted in a set of primers that enabled the detection of 100% of mutations in the panel.

Two sets of blinded validation experiments were performed, making this the most extensively validated HRM protocol described. Firstly, validation was performed by a prospective blinded screening of 266 samples sent in for diagnostic mutation testing using Sanger sequencing. An additional retrospective blinded validation was performed by screening 118 previously tested samples in which no mutations had been detected at the time of either full or partial testing. All previously described sequence variants could be detected and a pathogenic mutation in a previously untested region was detected.

HRM screening is most cost-effective in the absence of a large number of SNPs. The relatively low amount of SNPs in *BRCA1 *and *BRCA2 *make HRM a suitable mutation scanning technique for these genes. We used appropriate primer design (including the use of inosines) and placement to further minimise the SNPs within amplicons. Nevertheless those SNPs that occur within coding sequences could not be eliminated. While sequencing burden is significantly reduced via pre-sequencing HRM screening, approximately 7% of amplicons still require sequencing which is largely due to the detection of clinically non-significant polymorphisms. In our confirmatory sequencing, 97% of the identified sequences were SNPs, entailing an average of 7 amplicons that needed to be screened per patient.

Others have incorporated SNP genotyping using unlabelled probes as part of their protocol [37,38]. Although we have shown earlier that samples that are heterozygous for both a polymorphism and a mutation can be distinguished from those carrying a polymorphism alone, we believe that it is safer to sequence all sequence variants in a diagnostic scenario.

## Conclusions

Our protocol comprises 94 PCR amplicons that cover the complete coding region along with intron-exon boundaries of *BRCA1 *and *BRCA2*. The amplicons are designed to be run under the same PCR and HRM conditions. A 384 well format allows the greatest number of HRM assays to be performed at the one time as well as allowing more reliable detection of mutations. The closed-tube format used here has clear advantages over other screening technologies which require post-PCR product manipulation as there is a complete elimination of all risk of PCR product contamination along with considerable reduction in manual handling [8,39,40]. It was shown that the incorporation of deoxyinosine into the primers was useful in both allowing flexible primer placement and reducing the amount of subsequent sequencing necessary.

The protocol has applications within all diagnostic and research projects that have budget limitations. It can be run at a fraction of the cost of a full sequencing approach as the sequencing burden is relieved by more than 90%. This marked reduction in cost will enable *BRCA1 *and *BRCA2 *mutation detection to be more widely used especially for those who have a positive family history for breast or ovarian cancer but who do not meet current algorithms for mutation testing. The assay design principles in this paper are also relevant to the HRM screening of other germline mutations.

## Methods

### Patient DNA Samples

Samples and control DNA for the HRM optimisation and targeted mutation detection were obtained from the Diagnostic Molecular Pathology laboratory at the Peter MacCallum Cancer Centre and from the Kathleen Cunningham Foundation Consortium for Research into Familial Breast Cancer (kConFab). Samples for the blinded prospective validation (266) and for the blinded retrospective validation (118) were obtained from the Diagnostic Molecular Pathology laboratory at the Peter MacCallum Cancer Centre. Additional blinded *BRCA2 *mutation controls (18) and blinded DNA samples (10) were obtained from the Hunter Area Pathology Service, John Hunter Hospital and Royal Melbourne Hospital, respectively. This study was conducted under guidelines approved by the Peter MacCallum Ethics of Human Research Committee (approval number 03/90). All individuals had previously consented for germline *BRCA1 *and *BRCA2 *testing.

### Setting up of reactions

EpMotion 5075 (Eppendorf AG, Eppendorf, Germany) programs were designed for the dispensing of the mastermix and samples into pre-designated wells of a 384 well thermoplate (Roche Diagnostics, Penzberg, Germany). All reactions were set up in duplicate. Two alternative programs were used; a 6 plate program with 16 assays per plate screening 10 samples plus 1 wildtype control and 1 no-template control and a 12 plate program with 8 assays per plate screening 22 samples plus 1 wildtype control and a no-template control. The plate layouts are set out in Additional file 3 Table S1.

Following preparation of the mastermix, 7 uL was aliquotted into each pre-designated well. As multi-dispensing on the EpMotion is restricted to volumes equal or greater than 3 uL, the DNA (10 ng per reaction) was adjusted with PCR grade H_2_O to 3.3 ng/μl. The total time taken to set up each plate was less than 1 hour as mastermix dispensation and DNA addition each required 20 minutes.

### PCR and HRM conditions

Samples were run in duplicate using 10 ng of genomic DNA, 250 nM of forward and reverse primers, mastermix and PCR grade water in a total reaction volume of 10 uL. Either LightScanner Master Mix (Idaho Technology, Salt Lake City, UT) or TrendBio Master Mix (TrendBio Pty Ltd, Melbourne, Australia), supplemented by LCGreenPlus™ Hi-Res Melting Dye (Idaho Technology) were used. The primers for *BRCA1 *exons 11I, 11M, 17 and 18, and *BRCA2 *exon 16 were used at 500 nM final concentration. The primer sequences used are listed in Additional file 4 Table S2.

PCR and HRM was performed on the LightCycler 480 (Roche Diagnostics). Template amplification conditions included an activation step of 10 minutes at 95°C followed by 45 denaturation cycles of 95°C for 10 seconds, annealing for 10 seconds comprising 10 cycles of a touchdown from 65 to 55°C at 1°C/cycle followed by 35 cycles at 55°C, and extension at 72°C for 30 seconds. Prior to the HRM, a heteroduplex forming step involved heating the PCR products to 95°C for 1 minute and a rapid cooling to 45°C for 1 minute. HRM was performed from 72°C through to 95°C at a temperature gradient of 1°C per second, acquiring 30 data points per °C.

### HRM analysis

The melting curves were normalised at the pre-melt (100% fluorescence) and post-melt (0% fluorescence) stages using the supplied software temperature shifting [13] was used to compensate for temperature variation between wells and enabled samples with similar denaturation behaviour to be grouped. While there may be some loss of information with increasing temperature shifts, in particular, loss of detection of homozygous variations, visualisation of heterozygous profiles, particularly those of single base insertions and deletions is enhanced at greater temperature shifts. The default threshold used in our study was 5. However, the threshold can be lowered when an ambiguous sample is being examined. We used a default sensitivity setting of 0.70.

Samples with aberrant melting behavior were chosen as discussed in the Results section. A conservative approach was taken which maximises sensitivity at the loss of specificity i.e. some false positive calls were sequenced to avoid missing some of the more subtle mutations.

### Sequencing

Chosen samples were directly sequenced from a 1/35 dilution of the HRM product using the BigDye Terminator v3.1 cycle sequencing kit (Applied Biosystems, Foster City, CA). Following manual ethanol precipitation and clean up, Hi-Di™ Formamide (Applied Biosystems) denaturation, the samples were analysed on the ABI 3730 DNA sequencer (Applied Biosystems). The resulting sequence data was analyzed with Sequencher software, version 4.9 (Gene Codes, Ann Arbor, MI).

## Authors' contributions

HH carried out and analysed the molecular genetic studies and drafted the original manuscript. AD conceived the study, and participated in its design and coordination, took the manuscript to completion, and wrote the revision. AD and HH designed all the amplicons used in the final protocol. VB participated in the comparison of the blinded HRM assays with the sequencing results. SQW assisted in the data interpretation, analysis and in the writing of the revisions. SBF, GM and RJS participated in the design of the study and supply of specimens. kConFab supplied specimens with known mutations as positive controls. All authors read and approved the final manuscript.

## Pre-publication history

The pre-publication history for this paper can be accessed here:

http://www.biomedcentral.com/1471-2407/11/265/prepub

## Supplementary Material

Additional file 1 **Figure S1: Detection of mutations within a double melting domain**. Despite the very clear double melting domain, both mutations; *BRCA2 *c.6743del13bp (blue) and *BRCA2 *c.6821G>T (green) are readily differentiated from the wildtype (grey) in this 221bp amplicon. Click here for file

Additional file 2 **Figure S2: Visualisation of all three genotypes of the *BRCA1 *c.2612C>T SNP**. The wildtype C/C homozygote is distinct from the T/T homozygote. The heterozygote has a broader melting peak which is due to the combined melting peaks of the homoduplex and heteroduplex populations. Click here for file

Additional file 3 **Table S1: Reaction layout**. This table shows the layout used for each of the 12 plates in a 22 sample run. Each plate can run 8 assays with 22 test samples, a wildtype control and a no template control. Click here for file

Additional file 4 **Table S2: PCR primer sequences**. This table shows the final set of primers used. M13 sequences, where used, are indicated in bold. Click here for file

Additional file 5 **Table S3: List of known variants used in testing**. These represent the known mutations that were tested for *BRCA1 *and *BRCA2*. Click here for file

Additional file 6 **Table S4: List of previously untested variants found during validation**. These are the previously undetected variants that were detected during blinded testing. Click here for file
