# Supplementary figures and images for: A high-throughput protocol for mutation scanning of the BRCA1 and BRCA2 genes
Source: BMC Cancer. 2011 Jun 24;11:265. doi: 10.1186/1471-2407-11-265 (PMC3146935; doi:10.1186/1471-2407-11-265)

## Slide 1
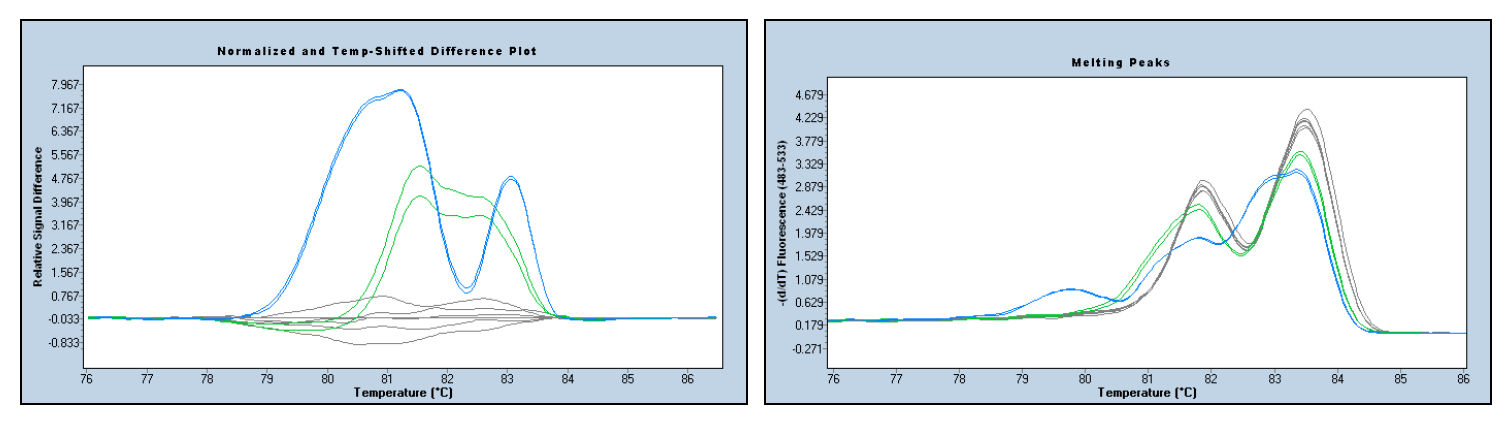

Supplement: Additional file 1 — Figure S1: Detection of mutations within a double melting domain. Despite the very clear double melting domain, both mutations; BRCA2 c.6743del13bp (blue) and BRCA2 c.6821G>T (green) are readily differentiated from the wildtype (grey) in this 221bp amplicon. [file 1471-2407-11-265-S1.PPT]

## Slide 1
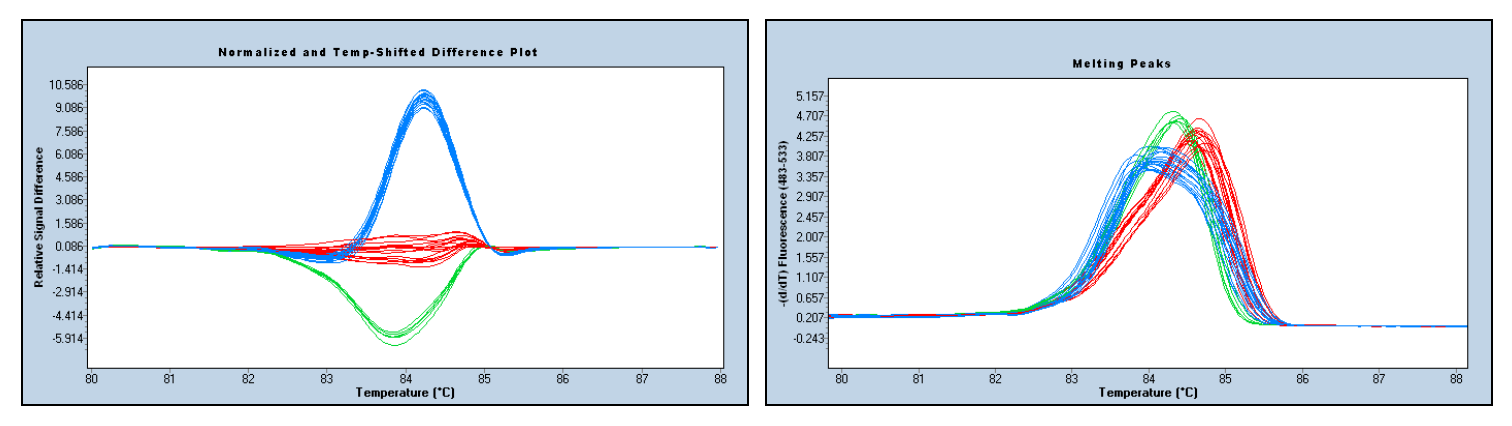

Supplement: Additional file 2 — Figure S2: Visualisation of all three genotypes of the BRCA1 c.2612C>T SNP. The wildtype C/C homozygote is distinct from the T/T homozygote. The heterozygote has a broader melting peak which is due to the combined melting peaks of the homoduplex and heteroduplex populations. [file 1471-2407-11-265-S2.PPT]
